# Alpha-1 antitripsyn deficiency and augmentation therapy in pregnancy: two case reports

**DOI:** 10.3389/fmed.2024.1479877

**Published:** 2024-12-16

**Authors:** Anna Annunziata, Giuseppe Fiorentino, Antonietta Coppola, Rosa Cauteruccio, Laura Ferrentino, Luigi Fiorentino, Cecilia Calabrese

**Affiliations:** ^1^Department of Intensive Care, Unit of Pathophysiology and Respiratory Rehabilitation, AORN Ospedali dei Colli, Naples, Italy; ^2^Department of Translational Medical Sciences, University Federico II, Naples, Italy; ^3^Department of Translational Medical Sciences, University of Campania ‘Luigi Vanvitelli’, AORN Ospedali dei Colli, Naples, Italy

**Keywords:** alpha-1 antitrypsin deficiency, pregnancy, augmentation therapy, emphysema, safety, bronchial asthma

## Abstract

Alpha-1 antitrypsin deficiency (AATD) is an inherited condition characterized by reduced plasma levels of alpha-1 antitrypsin (AAT), often leading to pulmonary diseases primarily emphysema and/or chronic obstructive pulmonary disease (COPD), but also bronchiectasis, bronchial asthma, or other less common disorders. Early diagnosis enables AAT augmentation therapy, which has proven to be effective in slowing down functional decline and improving survival rates. This article presents two cases of pregnant women with rare allelic variants of AATD who received AAT augmentation therapy, exploring the limited evidence on its safety during pregnancy and the potential role of decreased serum AAT levels in pregnancy-related complications.

## Introduction

Alpha-1 antitrypsin deficiency (AATD) is an inherited condition characterized by reduced levels of circulating alpha-1 antitrypsin (AAT) in plasma. Pulmonary involvement of AATD is heterogeneous, primarily associated with emphysema-dominant lung disease and/or chronic obstructive pulmonary disease (COPD). It can also present with bronchiectasis, bronchial asthma, or other less common disorders (1–3). Unfortunately, patients with AATD are often diagnosed late, at which point lung transplantation becomes the only viable treatment option (4). Conversely, when diagnosed earlier, weekly intravenous infusions of human AAT therapy are the only medical intervention capable of slowing the relentless decline in lung function and the progression of emphysema (5–7). In addition, a recent study showed the efficacy of AAT therapy in increasing the survival of affected patients (7).

Over the past decades, advancements in diagnostic techniques have enabled earlier detection of AATD. As a result, it is becoming increasingly common to diagnose the condition in women of childbearing age, and this trend is expected to rise in the near future. Currently, there is insufficient evidence regarding the safety of AAT replacement therapy during pregnancy, leaving young patients and their physicians to carefully weigh the potential risks and benefits of initiating or continuing treatment. This highlights the need for more research on this topic. In this paper, we present two clinical cases of women affected by AATD who underwent augmentation therapy during pregnancy. We compare these cases to the few existing reports in the literature and examine the potential implications of decreased serum AAT levels in pregnancy.

### Case 1 description

A 34-year-old woman, affected by severe bronchial asthma and AATD, Z/Mwhitestable genotype with AAT serum level of 57 mg/dL, was on treatment with AAT augmentation therapy at the dose of 60 mg/kg/week for approximately 2 years. The patient is regularly monitored at the Unit of Pathophysiology and Respiratory Rehabilitation, Monaldi Hospital, Naples (Table 1). The patient could not receive a biological treatment for severe asthma because her type-2 biomarkers, such as serum total IgE, blood eosinophils, and fractional exhaled nitric oxide, were under the cutoff values for the prescription. Despite receiving an optimized treatment for bronchial asthma that included high-dose inhaled corticosteroids, long-acting beta_2_ agonists, and long-acting muscarinic antagonists, and presenting spirometric parameters in the normal range [FVC 3.28 L (95%), FEV_1_ 2.70 L (89%), and FEV_1_/FVC 80%], AAT therapy was prescribed due to her history of frequent exacerbations and a progressive DLCO decline (dropping from 101 to 70% over 2 years), even though high-resolution computed tomography (HRCT) scans did not reveal any emphysema-like changes. We hypothesized that impairment in DLCO might occur before structural changes become evident in HRCT. While augmentation therapy was not typically indicated for our AAT-deficient asthmatic patient, a previous clinical case report provided support for our decision to proceed with this treatment (8). The patient showed no signs of hepatic involvement during her initial evaluation or throughout the follow-up period. AAT therapy was well tolerated, and she did not experience any asthma exacerbations or functional respiratory deterioration in the 18 months leading up to her pregnancy. Despite the limited evidence regarding the safety of AAT therapy during pregnancy, we opted to continue the augmentation therapy given the patient’s previous clinical improvement. The patient was closely monitored throughout the treatment for any adverse reactions or signs of intolerance. The pregnancy progressed without complications, resulting in the spontaneous delivery of a full-term newborn weighing 3,400 grams. Given the lack of literature regarding AAT therapy during breastfeeding and in alignment with the patient’s preferences, the augmentation treatment was temporarily halted for approximately 6 months. However, due to a subsequent slight decrease in DLCO to 68%, the therapy was reinitiated.

**Table 1:** Clinical features of the two AATD cases.

Case	Genotype	Phenotype	Baseline AAT (mg/dL)	6 months AAT (mg/dL)	Postpartum AAT (mg/dL)	Delivery (Week)	Weight at birth (Kg)	APGAR index
1	Z/Mwhitestable	Severe asthma with progressive DLCO decline	57	108	101	39	3.400	10
2	Q0Ourem/Q0Ourem	Emphysema	2	68	65	38	2.330	10

AADT, Alpha-1 antitrypsin deficiency.

### Case 2 description

**Figure 1:**
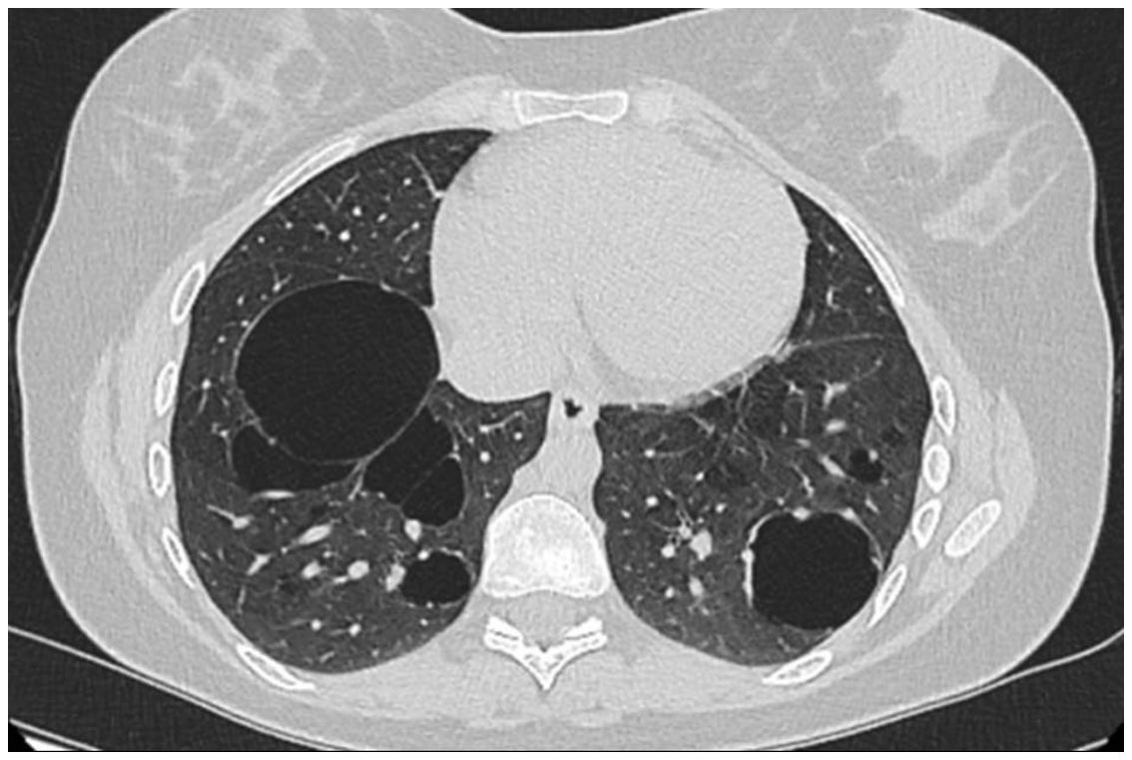
Chest HRTC of the patient with Q0Oreum/Q0Ourem genotype.

A 26-year-old non-smoking woman affected by AATD and bullous pulmonary emphysema, who has an undetectable AAT serum level and Null/Null genotype homozygous for Q0_Ourem_, has been receiving AAT augmentation therapy at a dose of 60 mg/kg/week for approximately 5 years. The patient is regularly monitored at the Unit of Pneumology Clinic, University of Campania Luigi Vanvitelli, Monaldi Hospital, Naples (Table 1). The diagnosis of AATD was established through family screening. The patient’s oldest brother had died at a young age from an unspecified lung disease, while another brother, a 31-year-old heavy smoker with severe COPD, was found to have no alpha-1 globulin fraction on serum protein electrophoresis, an undetectable serum AAT level, and DNA analysis confirmed a homozygous Q0Ourem genotype. Among the four sisters, only our patient exhibited a homozygous Q0Ourem genotype, while the others were heterozygous Q0Ourem. At the time of diagnosis, the patient reported only mild dyspnea following exercise. However, spirometry results revealed a decrease in both FEV1 (67%) and FVC (77%), while FEV1/FVC remained normal at 75%. In addition, there was a mild reduction in DLCO (67%). The 6-min walking test showed pathological results, with the patient’s peripheral oxygen saturation dropping below 88% after 5 min and covering 480 meters. Chest HRCT revealed pulmonary bullae in the lower lung lobes (Figure 1). A combination of long-acting β2 agonists and anticholinergics was prescribed alongside AAT augmentation therapy. The patient tolerated the treatments well, experiencing an improvement in exercise-induced dyspnea, with no further episodes of exercise-induced respiratory failure. In addition, there was a mild improvement in respiratory function parameters, with FEV_1_ increasing to 71%, FEV_1_/FVC to 81.5%, and DLCO to 74%. When the patient informed us of her pregnancy, a collective decision was made, in agreement with the patient, to continue only the augmentation therapy during gestation. The dual bronchodilator therapy was discontinued due to the observed improvement in both symptoms and respiratory function. The patient was closely monitored throughout her pregnancy, but unfortunately, she experienced a miscarriage during the second month. Six months later, she became pregnant again, and augmentation therapy with AAT was continued without interruption. No adverse reactions were observed, and the pregnancy progressed smoothly without any complications. Considering the presence of pulmonary bullae and after consulting with her gynecologist, a cesarean section was planned and successfully performed at 38 weeks of gestation. The patient delivered a healthy female infant weighing 2,330 grams. After delivery, a chest X-ray revealed bilateral sub-diaphragmatic emphysema, which was no longer evident on an HRCT scan of the thorax performed 1 month later. The patient decided to continue her AAT augmentation therapy and chose to feed her infant artificially.

## Discussion

AAT, a protein synthesized by the liver, was identified by Jacobsson in 1955, and the first cases of deficiency were reported in 1963 by Laurell and Eriksson (9). The primary function of AAT is to compensate for the activity of serine proteases, specifically neutrophil elastase, a proteolytic enzyme capable of causing structural alterations in the airways. In addition, AAT plays a crucial role in the immune system, modulating the balance between innate and adaptive immune responses and contributing to the development of immune tolerance (10–12). More recently, a potential role for AAT in human gestation has been proposed (12).

AAT levels typically increase by 4 to 6 times during pregnancy and gradually return to baseline pre-pregnancy levels in the postpartum period (13–15). Modifications in AAT levels and functions have been correlated with the regulation of fertility and a variety of pregnancy-related complications (11, 16–32). AAT plays a critical role in reducing inflammation in the placenta and regulating trophoblast apoptosis (11, 21). Consequently, AAT deficiency can heighten the inflammatory response, potentially impairing fetal development. Several studies have reported an increased risk of miscarriage in patients with AATD (11, 17, 18, 22), although Orimoloye et al. (28) observed that the likelihood of a previous miscarriage was higher specifically in mothers whose children were affected by AATD. Reduced levels of AAT have been observed in women with preeclampsia, identifying AATD as a potential risk factor for the condition (16, 17, 24). It has been shown that AAT expression varies in patients with pregnancy-induced hypertension, suggesting that AAT may contribute to the process of syncytialization of villous trophoblasts. In primary trophoblasts knocked down for AAT expression, some authors used RNA sequencing analysis to evaluate markers of syncytialization and inflammation. Their findings suggest that AAT dysfunction may lead to abnormal placental development, potentially contributing to pregnancy-associated disorders (29). Proteomic studies on amniotic fluid, vaginal fluid, or serum from women with preterm births have indicated that reduced AAT levels may serve as a biomarker for predicting preterm birth (17, 30). Specifically, low AAT levels detected during the first trimester have been identified as potential early indicators of preterm birth risk (31). Recent studies have shown that AAT modulates the neo-angiogenesis of small-diameter arteries. In pregnant patients, low AAT levels may interfere with the neoformation of small blood vessels in the uteroplacental circulation, leading to diminished fetal blood flow and impairing the fetal growth rate. This can result in low birth weight or infants whose weight is lower than expected for their gestational age (17, 28, 32). In the first population-based cohort study conducted in Denmark, Orimoloye et al. (28) examined 305 mothers and 254 children with AATD compared to controls. They found that AATD was associated with several pregnancy-related complications, including term low birth weight, children in the lowest quartile for abdominal circumference at birth (especially among non-smoking mothers), increased rates of cesarean delivery, preterm birth, and preeclampsia.

Given the potential adverse events associated with pregnancy in individuals with AATD, several authors have advocated for AATD testing in all pregnant women who have a history of miscarriages or preeclampsia, particularly those with known deleterious alleles. Beyond these pregnancy-related complications, various case reports have documented exacerbations of AATD-related conditions during pregnancy, including worsening respiratory symptoms, increased susceptibility to infections, pneumothorax, and panniculitis (22–26). In some cases, complications during pregnancy have led to the diagnosis of AATD (25, 26, 33). Diagnosis can be achieved through the examination of amniotic fluid and cord blood from unborn babies (34).

Augmentation therapy represents the only approved medical treatment for AATD, but its safety during pregnancy has not been tested in controlled studies. To date, there are only a limited number of case reports in the literature that describe the use of augmentation therapy during pregnancy in patients with AATD (8, 35, 36). Martínez-González et al. (35) reported a case of a 40-year-old woman, a former smoker with COPD and AATD (Pi*ZZ genotype), who was diagnosed at the age of 32. She continued her regular augmentation therapy throughout her pregnancy, which she tolerated well. At week 37 + 1, she underwent an elective cesarean section under epidural anesthesia, resulting in the delivery of a healthy infant weighing 2,400 grams, measuring 46 cm, with an APGAR score of 10/10. Gaeckle et al. (36) reported the case of a woman with AATD (Pi*ZZ genotype) who continued her augmentation therapy throughout her pregnancy. She tolerated the treatment well and experienced no complications. The baby was delivered at full term and, although born with a *patent ductus arteriosus*, was otherwise healthy. More recently, Guarnieri et al. (8) reported on a woman with AATD (Pi*SZ genotype) who had asthma and experienced a decline in both respiratory function and serum AAT levels during her pregnancy. At week 17, the patient suffered an asthma exacerbation, prompting the initiation of augmentation therapy. Despite experiencing another flare-up of bronchial asthma at week 28, the pregnancy progressed without complications. Similar to our case 1 report, the authors prescribed augmentation therapy to a patient with a deficient genotype and bronchial asthma, despite AAT therapy being primarily indicated for AATD patients with emphysema. Recent literature highlights an increased prevalence of asthma among AAT-deficient individuals, suggesting potential molecular mechanisms linking the two conditions, likely involving an imbalance between elastase and antielastase (37–39). Moreover, AAT deficiency, particularly Pi*S and Pi*Z SERPINA1 variants, has been associated with a higher risk of asthma exacerbations (40).

Consistent with the limited published cases, our patients tolerated AAT therapy during pregnancy without reporting any adverse effects. They both had full-term pregnancies and delivered healthy live infants. We hypothesize that AAT treatment may have contributed to the smooth progression of their pregnancies. While this paper is limited to just two cases of AAT therapy during pregnancy, and we acknowledge that the safety data may not be universally applicable to all patients with AATD, we believe it is crucial to share these clinical experiences. Due to the lack of scientific evidence regarding the safety of replacement therapy during breastfeeding, both of our patients opted for artificial feeding for their infants. Furthermore, while the existing clinical cases in the literature primarily focus on the more common ZZ and SZ genotypes (Table 2), to our knowledge this is the first report documenting AAT treatment in pregnant patients with the rare Z/M_whitestable_ and homozygous null Q0_Ourem_ genotypes.

**Table 2:** AATD clinical cases with pregnancy-related complications.

References	Genotype	Phenotype	AAT therapy	Duration of AAT therapy	Pregnancy-related adverse events
Giesler et al (22)	ZZ	COPD	No	–	Pulmonary infection requiring hospitalization
Atkinson et al (23)	No data	Emphysema	No	–	Pneumothorax
Dempsey et al (27)	ZZ	COPD	No	–	Respiratory symptoms at term, elected for cesarean section, with peri-procedure acute respiratory failure
Kennedy et al (24)	ZZ	No data	No	–	Intra-uterine growth retardation and pre-eclampsia
Kuller et al (25)	No S/Z	No data	No	–	Premature delivery and rupture of membranes, miscarriages, and spontaneous abortion
Yesudian et al (33)	ZZ	Panniculitis	No	–	Panniculitis
Martínez-González et al (35)	ZZ	Emphysema	Yes	9 years	No complications
Gaeckle et al (36)	ZZ	Emphysema	Yes	3 years	No complications
Guarnieri et al (8)	SZ	Asthma	Yes	Started during pregnancy	Mild asthma exacerbation
Furey et al. (26)	ZZ	Panniculitis	Yes	Started during pregnancy	Panniculitis

AAT, alpha-1 antitrypsin; COPD, chronic obstructive pulmonary disease.

## Conclusion

Reduced levels of AAT have been correlated with adverse pregnancy outcomes. However, the safety of augmentation therapy in pregnant women remains underexplored, with only a limited number of case reports available in the literature. Consequently, initiating or continuing AAT treatment during pregnancy presents a significant challenge. It is crucial to strongly advocate for the referral of pregnant women affected by AATD to national or regional expert centers. Furthermore, establishing dedicated registries and controlled clinical studies is essential to gathering more reliable data on the safety of AAT therapy, not only during pregnancy but also throughout the breastfeeding period. As diagnostic techniques improve, more patients will be screened and diagnosed with AATD at an earlier age. Consequently, an increasing number of young women with AATD, along with their physicians, will need to make informed decisions about whether to continue replacement therapy during pregnancy. Based on the available literature and the two cases we presented that highlight and support the safety and feasibility of AAT therapy during pregnancy, along with the potential adverse effects associated with AAT deficiency during this time, we advocate for the continuation of augmentation therapy in pregnant women with AATD.

## Data Availability

The raw data supporting the conclusions of this article will be made available by the authors, without undue reservation.

## References

[1] American Thoracic Society; European Respiratory Society. Thoracic society/European Respiratory Society statement: standards for the diagnosis and management of individuals with alpha-1 antitrypsin deficiency. Am J Respir Crit Care Med. (2003) 168:818–900. doi: 10.1164/rccm.168.7.818 14522813

[2] Aiello M Marchi L Ferrarotti I Frizzelli A Pisi R Calzetta L. Distribution of the clinical manifestations of alpha 1 antitrypsin deficiency in respiratory outpatients from an area of northern Italy. Respiration. (2022) 101:851–8. doi: 10.1159/000525549, PMID: 35793662

[3] Miravitlles M Dirksen A Ferrarotti I Koblizek V Lange P Mahadeva R. European Respiratory Society statement: diagnosis and treatment of pulmonary disease in alpha1-antitrypsin deficiency. Eur Respir J. (2017) 50:1700610. doi: 10.1183/13993003.00610-2017 29191952

[4] Verleden GM Gottlieb J. Lung transplantation for COPD/pulmonary emphysema. Eur Respir Rev. (2023) 32:220116. doi: 10.1183/16000617.0116-2022, PMID: 36948499 PMC10032585

[5] Chapman KR Burdon JG Piitulainen E Sandhaus RA Seersholm N Stocks JM. Intravenous augmentation treatment and lung density in severe α1 antitrypsin deficiency (RAPID): a randomised, double-blind, placebo-controlled trial. Lancet. (2015) 386:360–8. doi: 10.1016/S0140-6736(15)60860-1 26026936

[6] McElvaney NG Burdon J Holmes M Holmes M Glanville A Wark PA. Long-term efficacy and safety of α1 proteinase inhibitor treatment for emphysema caused by severe α1 antitrypsin deficiency: an open-label extension trial (RAPID-OLE) [published correction appears in lancet Respir med. 2017; 5(2): e13. Doi:10.1016/S2213-2600(17)30004-8]. Lancet Respir Med. (2017) 5:51–60. doi: 10.1016/S2213-2600(16)30430-1 27916480

[7] Fraughen DD Ghosh AJ Hobbs BD Funk GC Meischl T Clarenbach CF. Augmentation therapy for severe Alpha-1 antitrypsin deficiency improves survival and is decoupled from Spirometric decline-a multinational registry analysis. Am J Respir Crit Care Med. (2023) 208:964–74. doi: 10.1164/rccm.202305-0863OC, PMID: 37624745 PMC10870866

[8] Guarnieri G Achille A Lococo S Vianello A. Pregnancy in alpha 1 antitrypsin (AAT) deficiency and the role of intravenous AAT therapy. Pulmonology. (2022) 28:317–9. doi: 10.1016/j.pulmoe.2022.01.014, PMID: 35221261

[9] Laurell CB Eriksson S. The electrophoretic α1-globulin pattern of serum in α1-antitrypsin deficiency. 1963. COPD. (2013) 10:3–8. doi: 10.3109/15412555.2013.771956 23527532

[10] Guttman O Baranovski BM Schuster R Kaner Z Freixo-Lima GS Bahar N. Acute-phase protein a1-anti-trypsin: diverting injurious innate and adaptive immune responses from non-authentic threats. Clin Exp Immunol. (2015) 179:161–72. doi: 10.1111/cei.12476, PMID: 25351931 PMC4298394

[11] Jezela-Stanek A Chorostowska-Wynimko J. Beyond the lungs: alpha-1 antitrypsin’s potential role in human gestation. Adv Clin Exp Med. (2019) 28:1257–61. doi: 10.17219/acem/103668, PMID: 30929319

[12] Ehlers MR. Immune-modulating effects of alpha-1 antitrypsin. Biol Chem. (2014) 395:1187–93. doi: 10.1515/hsz-2014-0161, PMID: 24854541 PMC4237306

[13] Laurell CB. Orosomucoid and alpha 1-antitrypsin in maternaland fetal sera at parturition. Scand J Clin Lab Invest. (1968) 21:136–8. doi: 10.3109/00365516809084275 5706636

[14] Walker JE Campbell DM Ogston D. Blood levels of proteinase inhibitors in pregnancy. Br J Obstet Gynaecol. (1982) 89:208–10. doi: 10.1111/j.1471-0528.1982.tb03615.x 6175337

[15] Haram K Augensen K Elsayed S. Serum protein pattern in normal pregnancy with special reference to acute-phase reactants. Br J Obstet Gynaecol. (1983) 90:139–45. doi: 10.1111/j.1471-0528.1983.tb08898.x, PMID: 6600623

[16] Twina G Sheiner E Shahaf G Yaniv Salem S Madar T Baron J. Lower circulation levels and activity of a-1 antitrypsin in pregnant women with severe preeclampsia. J Matern Fetal Neonatal Med. (2012) 25:2667–70. doi: 10.3109/14767058.2012.705397 22746289

[17] Baron J Sheiner E Abecassis A Ashkenazi E Shahaf G Salem SY. a1-antitrypsin insufficiency is a possible contributor to preterm premature rupture of membranes. J Matern Fetal Neonatal Med. (2012) 25:934–7. doi: 10.3109/14767058.2011.600369, PMID: 21843112

[18] Madar T Shahaf G Sheiner E Brazg J Levinson J Yaniv Salem S. Low levels of circulating alpha-1 antitrypsin are associated with spontaneous abortions. J Matern Fetal Neonatal Med. (2013) 26:1782–7. doi: 10.3109/14767058.2013.801955, PMID: 23650930

[19] Salem SY Shahaf G Sheiner E Levinson J Baron J Madar T. Diminished activity of circulating a1-antitrypsin is associated with pre-gestational isolated obesity. J Matern Fetal Neonatal Med. (2015) 28:500–3. doi: 10.3109/14767058.2014.925442 24834800

[20] Greene DN Procter M Krautscheid P Mao R Lyon E Grenache DG. A1-antitrypsin deficiency in fraternal twins born with familial spontaneous pneumothorax. Chest. (2012) 141:239–41. doi: 10.1378/chest.11-0104 22215832

[21] Frochaux V Hildebrand D Talke A Linscheid MW Schlüter H. Alpha-1-antitrypsin: a novel human high temperature requirement protease A1 (HTRA1) substrate in human placental tissue. PLoS One. (2014) 9:e109483. doi: 10.1371/journal.pone.0109483, PMID: 25329061 PMC4203740

[22] Giesler CF Buehler JH Depp R. Alpha1-antitrypsin deficiency. Severe obstructive lung disease and pregnancy. Obstet Gynecol. (1977) 49:31–4. PMID: 299781

[23] Atkinson AR. Pregnancy and alpha-1 antitrypsin deficiency. Postgrad Med J. (1987) 63:817–20. doi: 10.1136/pgmj.63.743.817, PMID: 3502182 PMC2428537

[24] Kennedy SH Barlow DH Redman CW. Pre-eclampsia in a women with homozygous PiZZ alpha-1 antitrypsin deficiency. Case report. Br J Obstet Gynaecol. (1987) 94:1103–4. doi: 10.1111/j.1471-0528.1987.tb02298.x 3501313

[25] Kuller JA Katz VL McCoy MC Bristow C. Alpha 1-antitrypsin deficiency and pregnancy. Am J Perinatol. (1995) 12:303–5. doi: 10.1055/s-2007-994480 8540927

[26] Furey NL Golden RS Potts SR. Treatment of alpha-1-antitrypsin deficiency, massive edema, and panniculitis with alpha-1 protease inhibitor. Ann Intern Med. (1996) 125:699. doi: 10.7326/0003-4819-125-8-199610150-00027 8849167

[27] Dempsey OJ Godden DJ Martin PD Danielian PJ. Severe alpha1-antitrypsin deficiency and pregnancy. Eur Respir J. (1999) 13:1492–4. doi: 10.1183/09031936.99.13614959, PMID: 10445631

[28] Orimoloye HT He D Li T Janzen C Barjaktarevic I Wang X. Alpha-1 antitrypsin deficiency and pregnancy complications and birth outcomes: a population-based cohort study in Denmark. PLoS One. (2024) 19:e0296434. doi: 10.1371/journal.pone.0296434, PMID: 38166066 PMC10760838

[29] Yoshida K Yano A Kusama K Ishikawa G Tamura K. Alpha 1 antitrypsin regulates trophoblast Syncytialization and inflammatory factor expression. Int J Mol Sci. (2022) 23:1955. doi: 10.3390/ijms23041955, PMID: 35216073 PMC8879717

[30] Izumi-Yoneda N Toda A Okabe M Koike C Takashima S Yoshida T. Alpha 1 antitrypsin activity is decreased in human amnion in premature rupture of the fetal membranes. Mol Hum Reprod. (2009) 15:49–57. doi: 10.1093/molehr/gan071, PMID: 19073710

[31] D’Silva AM Hyett JA Coorssen JR. Proteomic analysis of first trimester maternal serum to identify candidate biomarkers potentially predictive of spontaneous preterm birth. J Proteome. (2018) 178:31–42. doi: 10.1016/j.jprot.2018.02.002 29448056

[32] Waugh JM Li-Hawkins J Yuksel E Cifra PN Amabile PG Hilfiker PR. Therapeutic elastase inhibition by alpha-1-antitrypsin gene transfer limits neointima formation in normal rabbits. J Vasc Interv Radiol. (2001) 12:1203–9. doi: 10.1016/s1051-0443(07)61680-7, PMID: 11585887

[33] Yesudian PD Dobson CM Wilson NJ. alpha1-antitrypsin deficiency panniculitis (phenotype PiZZ) precipitated postpartum and successfully treated with dapsone. Br J Dermatol. (2004) 150:1222–3. doi: 10.1111/j.1365-2133.2004.05991.x, PMID: 15214923

[34] Kaiser D Rennert O Goedde H Benkmann H-G Wuilloud H Kehrli P. Studies of amniotic fluid and cord blood in an infant with alpha 1-antitrypsin deficiency. Humangenetik. (1974) 25:241–5. doi: 10.1007/BF00281434, PMID: 4548687

[35] Martínez-González C Adanez Garcia J Blanco I. Term pregnancy of a patient with severe pulmonary emphysema associated with (PI*ZZ) alpha-1 antitrypsin. Embarazo a término de una paciente con enfisema pulmonar grave asociado a déficit grave (PI*ZZ) de alfa-1 antitripsina. Arch Bronconeumol. (2022) 58:427–8. doi: 10.1016/j.arbres.2022.02.004 35312536

[36] Gaeckle NT Stephenson L Reilkoff RA. Alpha-1 antitrypsin deficiency and pregnancy. COPD. (2020) 17:326–32. doi: 10.1080/15412555.2020.1754778, PMID: 32308050

[37] Pini L Paoletti G Heffler E Tantucci C Puggioni F Asthma and Alpha1-Antitrypsin Research Group. Alpha1-antitrypsin deficiency and asthma. Curr Opin Allergy Clin Immunol. (2021) 21:46–51. doi: 10.1097/ACI.0000000000000711 33284159

[38] Izquierdo M Rawal H Armstrong M Marion CR. Alpha-1 asthma overlap syndrome: a clinical overview. Curr Allergy Asthma Rep. (2022) 22:101–11. doi: 10.1007/s11882-022-01036-z, PMID: 35596100

[39] Annunziata A Lanza M Coppola A Fiorentino G. Alpha-1 antitrypsin deficiency in the elderly: a case report. J Med Case Rep. (2021) 15:231. doi: 10.1186/s13256-021-02847-w, PMID: 33966640 PMC8108364

[40] Martín-González E Hernández-Pérez JM Pérez JAP Pérez-García J Herrera-Luis E González-Pérez R. Alpha-1 antitrypsin deficiency and pi*S and pi*Z SERPINA1 variants are associated with asthma exacerbations. Pulmonology. (2023) 2023:2. doi: 10.1016/j.pulmoe.2023.05.002, PMID: 37236906

